# Going West

**Published:** 1857-09

**Authors:** 


### GOING WEST.

THAT is the rage now. Everybody is going west, or wants to go—except the wise ones. The "West" is a glorious country. It was the home of our childhood, and youth, and maturer years, and we love every foot of it; at the same time, it is not a "heaven" to everybody. Our personal experience capacitates us for making a safe judgment. While we have had every comfort and every convenience that many thousands could procure, we have known the exceeding comfort of a drink of spring water in a boundless waste, and the convenience of a saddle blanket for a covering, and a log for a pillow, the hardships "between times."

No married person, who can by close economy and steady industry make a moderate living, ought to think of emigrating after the age of forty years, except in very rare cases, for the following chief reasons:

1st. In a great many instances, you forego the advantages of good schools and a convenient attendance on Sunday worship.

2d. You abandon the old associations, and acquaintances, and friendships of earlier life, and go to form new ones in your old age, among people you never knew, and *never can know*, like those you left behind you: hence your attachments must be as uncertain as they are hazardous, and in both cases frail.

3d. You go "to get land for your children." If you want to pursue a course which shall most certainly insure them "success in life," teach them the efficiency of honorable industry, and, with your blessings on them, bid them go out and work for themselves as you did, and it will be a more enduring and a more elevating "fortune" to them, than if you could leave each child a thousand acres of the best land in the Mississippi valley.

4th. If you go "west" you will have more land, but you will have to work harder, and as long as you live. That harder work will never secure you the same domestic comforts and conveniences which you left behind you, to say nothing of your strugglings against *inevitable* sickness. Everybody will tell you that *his* neighborhood is healthy, but we do not believe that there is, to one born in the East, a single healthy spot, one yard square, between the *Alleghanies* and the *Sierra Nevada*. Any part of the "West" may become healthy to you in a few years, if you do not go to your grave in the mean time. As a private observer and a physician, we don't believe that any farm in the Mississippi valley is worth a "shake" of three years, *probably* seven.

An *Ignis Fatuus*, a kind of Jack-o'-Lantern delusion, possesses a man the moment the Western Mania seizes him. *His* acre here is worth two hundred dollars, and this value he transfers to his western farm. He soliloquizes thus: "My fifty acres here, will bring ten thousand dollars; that will purchase five hundred acres there, to be worth in a few years fifty thousand dollars," and at once he sees himself a millionaire. *In a few years?*—within that time a "farm" of six feet by two will be all that he can use.

Then again, there is a rating of the product of the soil there, at the retail price it brings here, leaving out of sight the ninety per cent. of profit that is swallowed up between the producing place and the market-house. Where we were "born and raised," the plenteous vision passes this moment before memory's eye, with associations mournfully pleasing. Eggs were brought to our father's bounteous table, by the peck, at "tuppence" a dozen; the largest turkeys at twenty-five cents a-piece; the best hams at three cents a pound, and prime beef-steak at four cents, the highest; the most splendid apples at fifty cents a barrel, and potatoes at twenty cents a bushel. Solid hickory wood cost, delivered, a dollar a cord; the best flour, two dollars a barrel, and others of the good things of life in the same proportion. Fresh grass butter went a-begging at ten cents a pound, and milk such as New York never sees, was fed to the pigs.

Things were cheap there for want of purchasers. Everybody had enough of his own. There were no beggars, and no loafers. All worked, and all had a plenty, as would be the case now under the same conditions. This blessed moment do we almost wish that we could be transported back again to those *good old times*, and to those plenteous places, a thousand miles from any railroad, and a week's wagon journey from any river in winter time.

We used to make many inquiries as to the clear gains of fine western farms and splendid southern plantations, for we have lived on and among both, and counting everything at a reasonable cash value, it was a rare occurrence, and it is *a rare occurrence still*, that any mere farmer or planter clears, above all expenses, over six per cent. on his entire capital and labor. Multitudes there are who have waited a lifetime to get rich by the "rise of property," and died poor at last, or the thriftless possessors of unproductive acres.

What tens of thousands of hearts have yearned their lives away for their eastern homes, but had not the means to return them there! These things are said of persons who have families, and are over forty years of age. To young persons, who have strong hands and willing hearts to work, and have nothing, we say, "*Go to the West.*"

But one of the greatest curses of the "West" is the mania for possessing land. The absorbing ambition is to possess the largest number of acres possible, and the next insanity is to have the widest extent of land under cultivation with the least labor. We have seen many a twenty acre field, which had scattered over it the labor due to one acre, with a less yield than the single acre well manured and thoroughly cultivated.

There are farmers in the very garden spot of Kentucky, embracing Bourbon, Scott, Clark, Fayette, Montgomery, and Woodford counties, who owned hundreds of acres of land that would produce, without manuring, a hundred bushels of corn to the acre, and yet, when they got to be sixty years of age, were only worth the land they lived upon.

Compare this with many a Dutch gardener around Cincinnati, who has grown rich in less than twenty years, by cultivating a few acres of side hill; or with a farmer in "Jersey," with its flat, sandy soil, which would not produce a mullein stalk or a jimson weed, without manure, who in eighteen hundred and fifty six "*cleared*," from twelve acres of land, on which he put two thousand dollars' worth of manure, yes *cleared*, above all expenses, seven thousand dollars! Such is his own statement.

Depend upon it, what we want in the East, as well as in the West, in order to make this country many times greater, more powerful, more productive, is,

First—Bring up more young men to the expectation of making a living by the cultivation of the soil.

Second—Let their expectation be to make a fortune by the product of the land, irrespective of any change in value.

Third—Teach them that one acre thoroughly cultivated, is more profitable than the same amount of labor spent on twenty acres.

Fourth—That to till the soil to the greatest advantage, the money which will yield the highest dividend by a hundred fold, is that expended in well-conducted agricultural periodicals. In short, that the requisites of successful farming are, *intelligence, liberal manuring, and thorough cultivation.*

